# The Proportion and Prognostic Significance of T-Regulatory Cells in Patients with Gynecological Cancers: A Systematic Review and Meta-Analysis

**DOI:** 10.7150/jca.42472

**Published:** 2020-03-05

**Authors:** Jiali Hu, Xirong Wu, Pengzhu Huang, Fei Teng, Yingmei Wang, Fengxia Xue

**Affiliations:** Department of Obstetrics and Gynecology, Tianjin Medical University General Hospital, 154 Anshan Road, He Ping District, Tianjin 300052, China.

**Keywords:** Gynecological cancer, T-regulatory cell, Proportion, Prognosis, Meta-analysis

## Abstract

**Objective**: Multiple reports have described the proportion of T-regulatory cells (Tregs) in peripheral blood (PB) and tissues in patients with gynecological cancers (GCs) with controversial results. Thus, the aim of this study was to investigate the proportion of Tregs and its prognostic survival role in GCs patients.

**Methods**: We performed a comprehensive search from database inception for all studies presenting changes of Tregs in GCs patients versus controls to evaluate the pooled standardized mean differences (SMD) with 95% confidence intervals (95% CI). And hazard ratios (HRs) with 95% CI were recorded if available to determine the prognostic significance of Tregs.

**Results**: Totally, 22 studies were included. Compared with controls, GCs patients had a higher proportion of Tregs in PB (SMD = 2.32, 95% CI = 1.47 to 3.17, *P* = 0.000) as well as in tissues (SMD = 3.47, 95% CI = 0.77 to 6.18, *P* = 0.012). Furthermore, more significant elevated frequency of Tregs was observed in GCs patients with advanced stage than those in the early stage in both PB and tissues. However, no association was found between Tregs and survival of GCs patients with an HR of 1.34 (95% CI = 0.96 to 1.88, *P* = 0.09).

**Conclusions**: Compared to controls, proportion of Tregs in PB and tissues was both higher among GCs patients, and it can be considered as a clinical biomarker for screening and prediction of clinical characteristics of GCs patients. But larger researches with rigorous design should be carried to explore the deep mechanisms of Tregs in GCs.

## Introduction

With roughly estimated 109,000 new cases and 33,100 deaths in 2019 in the United States, gynecological cancers (GCs) are considered as the fourth most frequent cancers in women nowadays, mostly including ovarian cancer (OC), endometrial cancer (EC), and cervical cancer (CC) 1. Therefore, more researchers were focusing on exploring more effective therapies and underlying mechanisms of GCs in order to improve patients' survival and alleviate economic burden on the national health care system 2-4. Tumor immunity, capacity of immune response to tumor, has aroused much attention with its undiscovered potential immunomodulatory properties on tumor progression 5. Undoubtedly, it also performed well in GC 6. T cells were the predominant kinds of immune cells which played vital roles in balancing tumor immune homeostasis between immune response and immune tolerance with respect to recent clinical developments of immunotherapies 7-9. T-regulatory cells (Tregs), a highly enriched T cells subset in tumor microenvironment, were considered to be important mediators resulting in the failure of human antitumor immune response in most kinds of cancers, such as breast cancer, liver cancer, and lung cancer 10-12.

However, answer to the question “whether Tregs perform as inhibitors or promoters in the development of GCs through shaping immunologic tolerance and ignorance” is still ambiguous. Some of the previous studies confirmed that patients with GCs had increased numbers of peripheral circulating and tumor infiltration Tregs, especially in those with advanced stages, high grades, poor differentiation, and unfavorable survival 13-19. In contrast, Saladin Sawan and colleagues reported fewer Tregs in patients with EC than benign controls 20. Further study also identified the accumulation of Tregs in tumor-draining lymph nodes from OC patients was lower than those from control nodes, and it presented less frequent in advanced stage (III and IV) as compared with early stage (I and II) surprisingly 21. Additionally, Ninke Leffers et al. added the reality that an increased number of Tregs indicated improved survival of OC patients 14. Generally, roles of Tregs in GCs have been a longstanding topic of debate which was complicated and controversial. Thus, we conducted a systematic review and meta-analysis aiming to evaluate the different proportion of Tregs between GCs patients and controls and discover its potential clinical and prognostic implications.

## Methods

### Search strategy and selection criteria

This analysis was conducted in accordance with the Preferred Reporting Items for Systematic Reviews and Meta-Analyses (PRISMA) Statement 22. An electronic search of the following databases from inception to June 25, 2019 was undertaken without language restrictions for studies in human of circulating and tumor infiltration Tregs in patients with GCs: PubMed, EMBASE, Web of Science, Cochrane Library, Scopus, SpringerLink, and ScienceDirect. The keywords of the search used were as follows: (“endometrial neoplasm” or “endometrial carcinoma” or “endometrial cancer” or “endometrium cancer” or “endometrium carcinoma” or “cancer of the endometrium” or “carcinoma of endometrium” or “uterine neoplasm” or “corpus uteri cancer” or “uterine cancer” or “uterine carcinoma”), (“ovarian cancer” or “carcinoma of the ovary” or “cancer of the ovary” or “ovarian carcinoma” or “ovarian neoplasms”), (“uterine cervical neoplasms” or “cervical cancer” or “carcinoma of the cervix” or “cancer of the cervix” or “cervical carcinoma” “cervical neoplasms”), and (“T-Lymphocytes, Regulatory” or “regulatory T cells” or Treg or CD4^+^CD25^+^FoxP3^+^ or CD4^+^CD25^+^). And searches on MeSH terms were added if available. Additionally, we also carefully scrutinized the reference lists of key publications to find all potentially relevant studies to broaden the scope of search. Since the study was not conducted on patients, no informed consent or ethical committee approval was needed.

### Inclusion and exclusion criteria

Inclusion criteria were as follows: (1) original studies; (2) researches on human; (3) full text can be found; (4) studies with a title or abstract including GCs and Tregs; (5) accessible proportion of circulating or tumor infiltration Tregs as mean ± standard deviation (SD) were evaluated using flow cytometry or immunohistochemical in GCs patients; and (6) availability of a hazard ratio (HR) and 95% confidence interval (95% CI) for survival. No limitation was applied for the subtype of GCs, severities of the cancers, disability level, as well as sex and race of the study subjects.

Excluded criteria were as follows: (1) reviews, case reports, conference abstracts, proposals, and letters to editors; (2) duplicate publications and overlapping data from different databases; (3) special unusual types of Tregs; (4) hematological malignancies since these tumors were derived from the immune cells; and (5) no sufficient data can be extracted for later evaluation.

Two reviewers evaluated the titles and assessed the full text of all articles independently to assess eligibility. Disagreement was resolved by consensus.

### Data extraction

The data and detailed information about the studies meeting the inclusion criteria were extracted by two independent reviewers via a predefined data extraction form. And quality of the eligible studies was evaluated based on the Newcastle-Ottawa Quality Assessment Scale (NOS) including three parameters: selection, comparability, and exposure 23. The predefined data extraction form included name of the first author, year of publication, country of the study, types of GCs, numbers and mean age of GCs patients and controls, International Federation of Gynecology and Obstetrics (FIGO) stage or clinical stage, pathologic grade, sources of samples, detection methods, the definitions of Tregs used, and scores of NOS. Importantly, the proportion of circulating and tumor infiltration Tregs was recorded clearly. And HR was extracted preferentially from multivariable analyses when available. Otherwise, HR from univariate analyses was extracted. Corresponding authors were contacted to clarify any missing and ambiguous data.

### Statistical analysis

Stata version 12 software was employed to compute calculations and prepare graphs. We assessed the status of Tregs in the peripheral blood (PB) and tissues of patients with GCs as continuous outcomes, and calculated pooled estimates of the standardized mean differences (SMD) with 95% CI of the proportion of Tregs to present its difference between GCs patients and controls. Additionally, pooled HR with 95% CI was computed and weighted using generic inverse-variance to evaluate the prognostic significance of Tregs in GCs patients. Chi-squared Q test and *I^2^* statistics were used to assess heterogeneity. When *P* < 0.1 or *I^2^* > 50%, the heterogeneity was considered significant moderate-to-high and a random effect model was used. Otherwise, a fixed effect model was used. Subgroup analysis and sensitivity analysis were carried out to investigate the potential effects of study characteristics and certain single study that may influence the final results. Possibility of publication bias was assessed by constructing a funnel plot whose asymmetry was later evaluated using Begg's and Egger's tests to determine each study's effect against standard error.* P* < 0.05 was considered significant.

## Results

### Study characteristics

**Figure 1:**
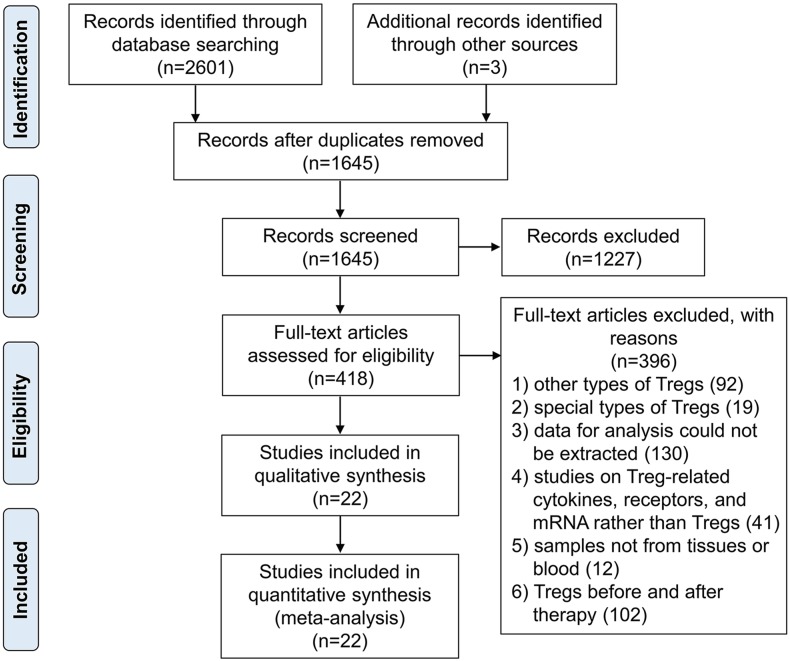
The flow chart of the article search and inclusion process following the PRISMA guidelines.

The flow chart of the article search and inclusion process was detailed in Figure 1. Base on this search strategy, we identified 2604 studies, of which 22 studies were included in the final meta-analytical processes involving 2115 GCs patients and 470 controls. Main characteristics of the included studies were listed in Table 1. All studies were retrospective researches including 12 of OC, 6 of CC, and 4 of EC. The recruitment of most studies (14 studies) were consecutive with the remainder being unknown. Average NOS score of the included studies was 6.91 (range from 5 to 9). Samples from PB and tissues were mostly tested by flow cytometry and immunohistochemistry.

### The proportion of Tregs in GCs patients

**Figure 2:**
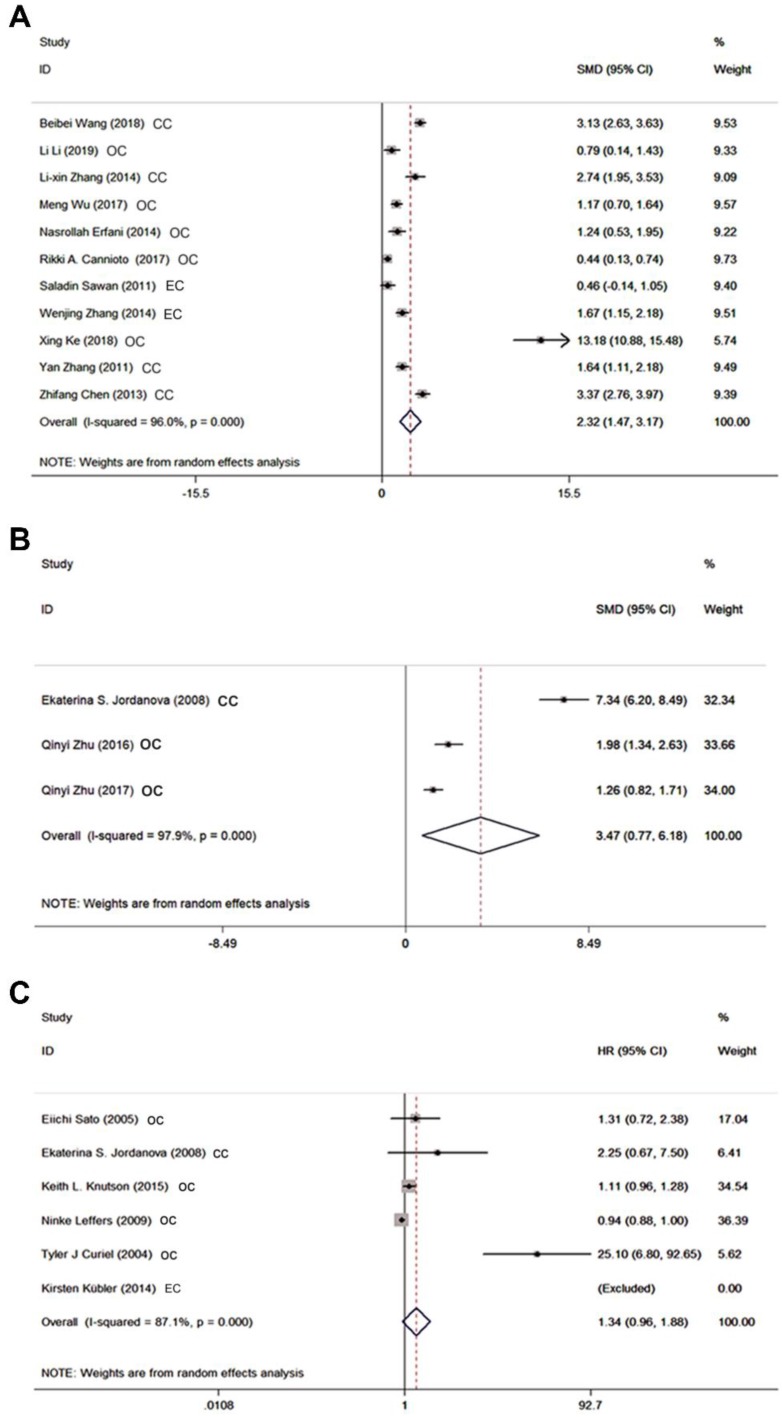
Forest plots showing the association between Tregs and GCs patients. **A** SMD of Tregs proportion in PB between GCs patients and controls. **B** SMD of Tregs proportion in tissues between GCs patients and controls. **C** HR for survival of Tregs in tissues greater than or less than the cutoff value.

We initially compared the proportion of circulating Tregs in GCs patients with controls in 11 studies regardless of what kind of Tregs definitions had been used. Results in Figure 2A revealed that GCs patients had significantly increased frequency of Tregs in PB with SMD of 2.32 (95% CI = 1.47 to 3.17, *P* = 0.000). Since there was statistically significant heterogeneity among studies (*I^2^*= 96%), random effect model was applied. Additionally, high abundance of Tregs was proved to be associated with advanced FIGO stage for the SMD of advanced stage versus early stage was 0.45 (95% CI = 0.02 to 0.87, *P* = 0.038). As for the results of tissues, pool analysis of three studies showed there was also a significant increased proportion of tumor infiltration Tregs in GCs patients when compared with controls [SMD 3.47 (95% CI = 0.77 to 6.18, *P* = 0.012) (Figure 2B). And similar to the results in PB, a slight increase was observed when compared tumor infiltration Tregs in GCs patients on advanced stage with those on early stage (SMD = 0.53, 95% CI = 0.25 to 0.81, *P* = 0.000).

### The prognostic value of Tregs on survival in GCs patients

Six studies comprising 1119 patients were focused on results of Tregs in tissues which reported HR with 95% CI for survival involving overall survival, disease-specific survival, and tumor associated survival. When we analyzed the prognostic significance of Tregs in GCs patients all together, the pooled HR was 1.34 (95% CI = 0.96 to 1.88, *P* = 0.09) indicating their incapacity to predict the prognosis of GCs patients (Figure 2C). And four studies out of six all evaluated overall survival. In this condition, we also found no statistically significant association between tumor infiltration Tregs and GCs according to the pooled HR of 1.13 (95% CI = 0.98 to 1.30, *P* = 0.08).

### Subgroup analysis

Subgroup analysis was performed to explore the impact of presumptive potential factors including types of GCs, score of NOS, and definitions of Tregs, that may influence the final results (Table 2). Due to the limited studies of tissues, we only conducted subgroup analysis of studies in PB in detail. Similar to its general role in GCs, accumulation of circulating Tregs were also observed high in patients with OC and CC respectively, with SMD for OC 2.64, 95% CI = 1.20 to 4.08 and SMD for CC 2.72, 95% CI = 1.90 to 3.53. However, no statistical significance was found in EC with SMD of 1.07 (95% CI = -0.11 to 2.25). When classified by NOS score, both subgroups presented high proportion of Tregs in GCs patients. Although different definitions of Tregs based on diverse markers may influence the pooled SMD, most kinds of definitions listed in Table 2 showed elevated numbers of circulating Tregs in GCs patients when compared to controls except CD4^+^FoxP3^+^.

### Publication bias

**Figure 3:**
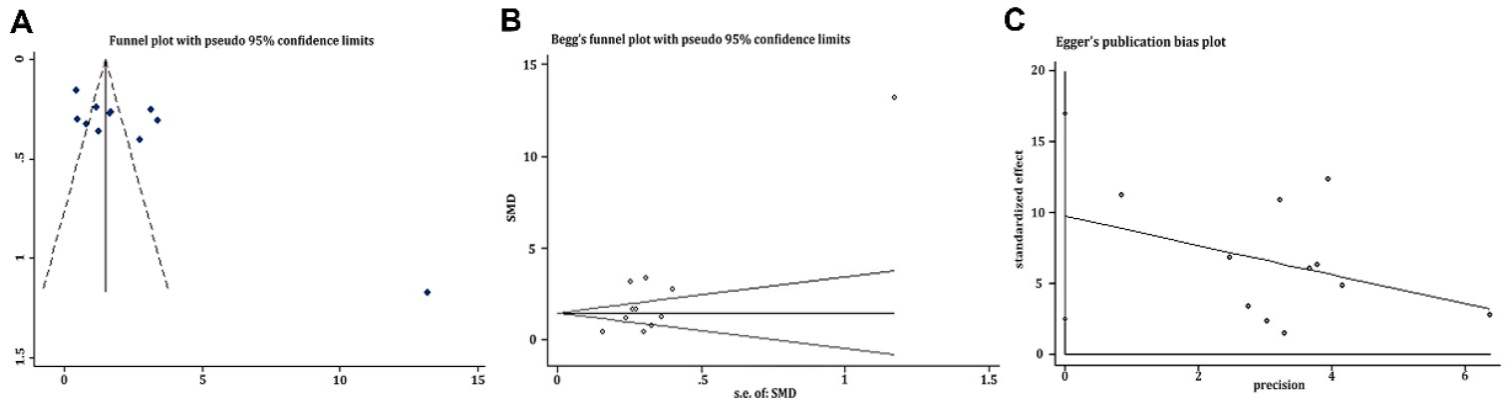
Evaluation of potential publication bias of the included researches on Tregs in PB. **A** Funnel plot. **B** Begg's funnel plot. **C** Egger's publication bias plot.

Funnel plot was depicted to describe the publication bias of researches on circulating Tregs, which showed slight significant asymmetry generally in Figure 3. That is to say, publication bias was not controlled well enough here. And results of Begg's test and Egger's test presented a consistent trend with what showed in funnel plot with both *P* = 0.014 as well as asymmetric figures (see in Figure 3). Thanks to only three studies focusing on Tregs in tissues and only six studies aiming at the evaluation of HR, we didn't draw funnel plots of these issues.

### Sensitivity analysis

**Figure 4:**
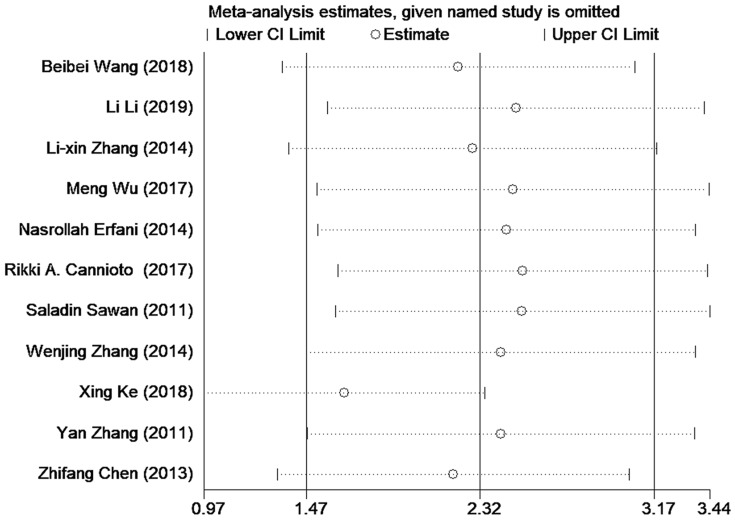
Sensitivity analysis of the included researches on Tregs in PB.

Sensitivity analysis was conducted to explore the potential study that may contribute to data heterogeneity by omitting studies one by one. And no significant changes in the results were found except for excluding the study of Xing Ke (see in Figure 4).

## Discussion

### Proportion of Tregs in PB was a biomarker for GCs

Though there were substantial strides forward in the general understanding of Tregs that an elevated accumulation of it contributed to the development of some cancers, no consistent explicit roles of Tregs in GCs have been determined by previous reports yet 11,12,37. That is to say, status of Tregs in GCs patients was still under debate. Therefore, we undertook a meta-analysis of 22 studies compromising three major types of GCs with 2115 patients to elucidate the clinical implications and prognostic value of Tregs in GCs. Our findings saw a consistent trend of increased frequency of Tregs in both PB and tissues which agreed with the phenomena that elevated accumulation of Tregs had the ability to hamper effective anti-tumor immune responses and maintain immunological tolerance in tumor-bearing hosts via co-operative interaction with certain other immune cells 38,39. Of interest, frequency of Tregs in tissues was found mildly higher than those in PB (3.47 vs 2.32) for the reason that intra-tumoral Tregs originated primarily from certain kind of PB Tregs which were proved to be inclined to move into the lesion areas with the stimulation of inflammatory factors contributing to the progression of cancers 40. And a study by Wu et al. also reported that imbalance of Tregs in the tumor microenvironment influenced the energetic metabolic processes including increased glucose uptake and fermentation of glucose to lactate, which had an important role in controlling cancer initiation and progression 41. Therefore, no wonder proportion of Tregs within tumors presented at a higher level than those in PB, which drove a state of immune disorders to promote the occurrence of GCs. Additionally, the phenomena that proportion of Tregs in GCs patients with advanced stage presenting higher than those with early stage, suggested the potential role of Tregs as a clinical biomarker to indicate poor prognosis which may help to aid patient stratification and tailor therapy for GCs patients.

### Prognostic value of tumor infiltration Tregs needed more investigations

Relationship between survival of GCs patients and tumor infiltration Tregs was observed negative in general whatever on the basis of the ratio of Tregs/CD4^+^ lymphocyte or Tregs/lymphocytes in this article. This was inconsistent with the conclusion drew by Shang B et al. that Treg infiltration was significantly associated with shorter overall survival in the majority of solid tumors, including cervical cancer 42. The reason for this inconformity might lie in the fact that we analyzed HR rather than the odds ratio to evaluate the prognostic role of Tregs. The included literatures in Shang B et al.'s research and ours were also not the same. And HR was preferred to be applied in many survival analyses for it considered the time factor 43. In this present meta-analysis, we hypothesized that the unexpected negative result might be influenced by the following reasons. Firstly, combined analysis might be highly influenced by different tumor site, severities of the disease, molecular subtype, and tumor stage 17,28,36. Secondly, results of survival analysis were based on measurement of Tregs in tissues through immunohistochemistry which provided accurate positions but offered no exact total amount and ratio of Tregs 13,27,30. Actually, flow cytometry was preferred under this condition, and it was more suitable to determine the cutoff values. And finally, included studies applied different definitions to identify Tregs leading to the insignificant difference in survival affected by Tregs 14,29,30. Therefore, a correct and reasonable definition was expected to contain not only the classical distinct markers but also some markers to identify the biological function of Tregs. Additionally, values of cutoffs also played a vital role in determining the significance of Tregs in GC patients' survival. Of important, although cutoffs values of Tregs ratio determined mostly by the median values of immunostaining was reported to divide tumors into high and low frequency of Tregs group, the specific methods of how to group remained vague 13,14,21,27,29,30. Thus, it was urgent and important for us to set up more well-designed and broader spectrum of subjects joined researches to clarify this issue.

### Subgroup analysis

Summarized from the results of subgroup analysis, we found some interesting phenomena. Compared with controls, patients with CC and OC possessed a high proportion of Tregs in PB. Conversely, such situation didn't occur in those with EC [SMD 1.07 (-0.11, 2.25)]. It was the limited total two of the included studies evaluating Tregs in EC that may cause this negative result 20, 28. Additionally, frequency of Tregs in PB was always reported to present a high trend in patients with GCs in spite of classification by different scores of NOS of studies. Moreover, the proportion of circulating Tregs identified by different definitions was all proved to be higher in GCs patients versus those without except for the definition as CD4^+^FoxP3^+^ cells. But, most of the included researches applied CD4^+^CD25^+^FoxP3^+^ not CD4^+^FoxP3^+^ as the standard criterion to identify Tregs in this meta-analysis. How to define Tregs could be the key point to influence the results. Multiple reports had already described differentiative and functional properties of Tregs were dependent on the expression of the FoxP3, and consequently, FoxP3 was considered as the key intracellular molecule and specific marker for Tregs so far 44, 45. While other opposite voices declared that FoxP3 couldn't be an exclusive marker for Tregs, since it was also upregulated in other activated immune cells 46. Thus, it was significant for us to discover additional appropriate and precise markers to distinguish Tregs from other immune cells correctly to enhance the reliability of further studies. Besides, subgroup analysis of data on tissues infiltration Tregs was not performed because the included researches examining this ratio were too scarce.

### Publication bias and sensitivity analysis

Results of Begg's test and Egger's test based on data of Tregs in PB both suggested there was some publication bias which reminded us to interpret the final results with caution. This might be attributed to inclusion of small sample researches in this study. And positive results were more easily to be published than negative ones. Therefore, further studies with a larger spectrum of patients ought to be carried out, and those with negative results should be encouraged to be published. Additionally, the picture of sensitivity analysis demonstrated certain stabilization of our pooled results and the relatively high heterogeneity of pooled SMD was possibly due to study by Xing Ke. Therefore, we omitted this study to find that heterogeneity decreased from 96.0% to 94.1%, and pooled SMD still had statistical meaning [SMD = 1.65 (95% CI = 0.97 to 2.34), *P* =0.000]. This phenomenon confirmed the reliability of our primary results.

### Limitations

Although we believed that the current meta-analysis provided some useful information, there were still some potential limitations should be addressed. Firstly, only summarized data rather than individual patient's data could be used. Secondly, heterogeneity in our study was substantial. So, it was cautious for us to interpret the results based on evaluation via a random effect model. Thirdly, we only included studies reporting on the values of SMD and HR, and consequently enormous publications reporting on the clinical and prognostic value of Tregs as odd ratios and relative risks were excluded. Fourth, proportion of Tregs in PB and tissues was nonspecific parameters, which may be influenced by concurrent conditions such as infections, inflammation, and medication, resulting in the confusion of Tregs' measurement. Fifth, we could not conduct subgroup analysis of different level of age, weight, pathologic grade, and tissues due to lack of sufficient original data from the included studies.

## Conclusion

Generally, our findings clearly lent support to the theory that Tregs was a promising biomarker to distinguish patients with GCs from healthy controls and it also possessed the ability to indicate the clinical characteristics of patients. And independent cohorts of patients with a larger spectrum of patients and controls are expected to validate our results forcefully.

## Figures and Tables

**Table 1:** Characteristics of the included studies

First author and year	Country	Types of GCs	PN	Age of Patients	CN	Age of Controls	FIGO/Clinical stage	Pathologic grade	Source of samples	DM	Definitions of Tregs	NS	Refs
Ekaterina S. Jordanova 2008	UK	CC	115	48.5 (24-87)	9	46 (31-60)	IB1 (55), IB2/II (60)	NA	Tissues	QFEI	FoxP3^+^	8	13
Walayat Shah 2011	China	CC	40	47 (32-70)	NA	NA	II (10), III (30)	NA	Tissues	IHC	CD4^+^FoxP3^+^	5	19
Yan Zhang 2011	China	CC	49	44 (34-70)	28	42 (26-67)	I (34), II (15)	G1 (8), G2 (19), G3 (22)	PBMCs	FC	CD4^+^CD25^+^FoxP3^+^	8	17
Zhifang Chen 2013	China	CC	65	45.50 ± 6.12	40	45.35 ± 6.17	I (26), II (39)	G1 (11), G2 (21), G3 (33)	PBMCs	FC	CD4^+^CD25^+^FoxP3^+^	9	24
Li-xin Zhang 2014	China	CC	30	NA	20	NA	NA	NA	PBL	FC	CD4^+^CD25^+^FoxP3^+^	5	25
Beibei Wang 2018	China	CC	70	50.5 ± 11.61	70	48.8 ± 9.5	I (9), II (45), III (16)	NA	PB	FC	CD4^+^CD25^+^	8	26
Saladin Sawan 2011	UK	EC	24	66 (44-92)	21	44 (35-80)	I (13), II (4), III (7)	G1 (7), G2 (5), G3 (6)	PBMCs	FC	CD4^+^FoxP3^+^	8	20
Wataru Yamagami 2011	Japan	EC	53	58 (39-81)	NA	NA	I (23), II (4), III (23), IV (3)	G1 (25), G2 (13), G3 (12), Others (3)	Tissues	IHC	CD4^+^FoxP3^+^	6	16
Kirsten Kübler 2014	Germany	EC	163	68 ± 10.37	NA	NA	I (128), II (17), III (12), IV (6)	G1 (15), G2 (114), G3 (34)	Tissues	IHC	FoxP3^+^	5	27
Wenjing Zhang 2014	China	EC	64	55 (31-80)	26	45 (26-67)	I (50), II (5), III-IV (6), Unknown (3)	G1 (30), G2 (15), G3 (10), Unknown (9)	PBMCs	FC	CD4^+^CD25^+^FoxP3^+^	8	28
Tyler J Curiel 2004	USA	OC	70	63.2 (39-77)	5	NA	I (7), II (7), III (41), IV (15)	G1 (7), G2 (8), G3 (55)	Tissues	IFT	CD3^+^CD4^+^FoxP3^+^	7	21
Eiichi Sato 2005	Japan	OC	117	62 (33-89)	NA	NA	I (5), II (7), III (91), IV (12), NA (1)	G1 (8), G2 (4), G3 (105)	Tissues	IHC	CD25^+^FoxP3^+^	6	29
Ninke Leffers 2009	Netherlands	OC	306	57.2 ± 13.5	NA	NA	I (67), II (24), III (171), IV (42), NA (2)	G1 (52), G2 (80), G3 (135), UD (14), Missing (25)	Tissues	IHC	FoxP3^+^	6	14
Jason C. Barnett 2010	USA	OC	232	58 (19-88)	NA	NA	I (24), II (13), III (127), IV (27), Unknown (2)	Borderline (39), G1 (20), G2 (90), G3 (83)	Tissues	IHC	FoxP3^+^	6	15
Nasrollah Erfani 2014	Iran	OC	17	50.3 ± 11.6	20	49.8 ± 8.0	I (3), II (3), III (8), IV (3)	NA	PBMCs	FC	CD4^+^CD25^+^FoxP3^+^	9	18
Keith L. Knutson 2015	USA	OC	348	63 (28-86)	NA	NA	I (41), II (15), III (265), IV (84)	G1 (10), G2 (393), G3 (2)	Tissues	IHC	CD4^+^CD25^+^FoxP3^+^	5	30
Qinyi Zhu 2016	China	OC	40	NA	20	NA	I (11), II (9), III (19), IV (1)	G1 (1), G2 (18), G3 (21)	Tissues	IFT	CD4^+^FoxP3^+^	5	31
Meng Wu 2017	China	OC	61	48.22 ± 9.60	30	NA	I-II (12), III-IV (49)	NA	PBMCs	FC	CD4^+^CD25^+^FoxP3^+^	8	32
Qinyi Zhu 2017	China	OC	126	Mean = 51.4	26	Mean = 52.15	I (34), II (30), III (61), IV (1)	G1 (12), G2 (37), G3 (77)	Tissues	IFT	CD4^+^FoxP3^+^	6	33
Rikki A. Cannioto 2017	USA	OC	71	58.1 ± 11.0	101	57.2 ± 10.9	NA	NA	PBMCs	FC	CD3^+^CD4^+^CD25^+^FoxP3^+^	7	34
Xing Ke 2018	China	OC	34	56.3 ± 6.8	34	51.8 ± 5.2	I-II (18), III-IV (16)	G1-G2 (20), G3 (14)	PBMCs	FC	CD4^+^CD25^high^CD127^low^	8	35
Li Li 2019	China	OC	20	45.5 ± 7.8	20	44.5 ± 6.1	I-II (6), III-IV (14)	NA	PBMCs	FC	CD4^+^CD25^+^CD127^-^CXCR5^+^FoxP3^+^	9	36

PN: number of patients; CN: number of controls; DM: detection methods; NS: scores of NOS; Ref: references; NA: not available; QFEI: quadruple fluorescent and enzymatic immunostaining; IHC: immunohistochemistry; PBMCs: peripheral blood mononuclear cells; FC: flow cytometry; PBL: peripheral blood lymphocyte; IFT: immunofluorescence technique.

**Table 2:** Subgroup analysis of SMD of Tregs in PB

Subgroup	No. of studies	SMD (95% CI)	Overall effect *P* value	Test of heterogeneity	
				*I^2^*	*P* value
Types of GCs					
OC	5	2.64 (1.20, 4.08)	*P* = 0.000	96.7%	*P* = 0.000
CC	4	2.72 (1.90, 3.53)	*P* = 0.000	86.7%	*P* = 0.000
EC	2	1.07 (-0.11, 2.25)	*P* = 0.076	88.9%	*P* = 0.003
Scores of NOS					
≥ 7	10	2.28 (1.38, 3.18)	*P* = 0.000	96.3%	*P* = 0.000
< 7	1	2.74 (1.95, 3.17)	*P* = 0.000	NA	NA
Definitions of Tregs					
CD4^+^CD25^+^	1	3.13 (2.63, 3.63)	*P* = 0.000	NA	NA
CD4^+^FoxP3^+^	1	0.46 (-0.14, 1.05)	*P* = 0.131	NA	NA
CD4^+^CD25^+^FoxP3^+^	6	1.95 (1.27, 2.64)	*P* = 0.000	87.8%	*P* = 0.000
CD3^+^CD4^+^CD25^+^FoxP3^+^	1	0.44 (0.13, 0.74)	*P* = 0.005	NA	NA
CD4^+^CD25^+^CD127^-^CXCR5^+^FoxP3^+^	1	0.79 (0.14, 1.43)	*P* = 0.017	NA	NA
CD4^+^CD25^high^CD127^low^	1	13.18 (10.88, 15.48)	*P* = 0.000	NA	NA

NA: not available
